# Effects of pectin liquid on gastroesophageal reflux disease in children with cerebral palsy

**DOI:** 10.1186/1471-230X-8-11

**Published:** 2008-04-16

**Authors:** Reiko Miyazawa, Takeshi Tomomasa, Hiroaki Kaneko, Hirokazu Arakawa, Nobuzo Shimizu, Akihiro Morikawa

**Affiliations:** 1 Department of Pediatrics and Developmental Medicine, Gunma University Graduate School of Medicine, Maebashi, Gunma, Japan; 2 PAL Clinic, Isesaki, Gunma, Japan; 3 MIRAI Kids Clinic, Midori, Gunma, Japan; 4 Gunma Rehabilitation Centre for the Physically Handicapped Children, Takasaki, Gunma, Japan

## Abstract

**Background:**

The use of thickeners is a standard therapy for decreasing episodes of regurgitation or vomiting in infants. However, it remains to be investigated whether thickener is effective for vomiting and/or chronic respiratory symptoms in children with cerebral palsy.

**Methods:**

We enrolled 18 neurologically impaired children caused by cerebral palsy, with gastroesophageal reflux disease. In the first part of this study (pH monitoring), subjects were randomly allocated to two groups: fed with a high-pectin diet [enteral formula: pectin liquid = 2:1 (v/v)], or a low-pectin diet [enteral formula: pectin liquid = 3:1 (v/v)]. Two-channel esophageal pH monitoring was performed over 48 h. In the second part (clinical trial), subjects were fed a high- or low-pectin diet and non-pectin diet for 4 weeks in a crossover manner. Nurses recorded the feeding volume, number of episodes of vomiting, volume of gastric residue, episodes of cough and wheeze, frequency of using oxygen for dyspnea, and the day when the child could return to school. Cough and wheeze were recorded as a cough-score.

**Results:**

The median value for the % time pH < 4 at the lower and upper esophagus was significantly decreased with a high-pectin diet [9.2% (6.2–22.6) vs. 5.0% (3.1–13.1); P < 0.01, 3.8% (2.9–11.2) vs. 1.6% (0.9–8.9); P < 0.01 (interquartile range), non-pectin and high-pectin, respectively]. The number of reflux episodes per day and duration of longest reflux were decreased significantly with a high-pectin, but not with a low-pectin diet. The median number of episodes of vomiting decreased significantly with a high-pectin diet [2.5/week (1.0–5.0) vs. 1.0 (1.0–1.5), P < 0.05]. The median cough-score was significantly decreased by both concentrations of pectin [8.5/week (1.0–11.5) vs. 2.0/week (0.0–3.0), fed with a high-pectin diet; 7.0/week (1.0–14.5) vs. 1.0/w (0.0–5.0), fed with a low-pectin diet, P < 0.05].

**Conclusion:**

Pectin liquid partially decreased gastroesophageal reflux as measured by eshophageal pH monitoring, and might improve vomiting and respiratory symptoms in children with cerebral palsy.

**Trial registration:**

ISRCTN19787793

## Background

Gastroesophageal reflux (GER) appears to be a common, persistent, and severe disorder in children with neurologic impairment [1-3]. The estimated incidence of GER in patients with cerebral palsy ranges from 32 to 75% [4,5]. Reflux episodes not only cause gastrointestinal symptoms, such as episodes of regurgitation or vomiting, haematemesis, and reflux esophagitis, but also respiratory problems, such as recurrent respiratory infections, persistent cough, life-threatening apneic episodes, and respiratory failure during fairly minor respiratory infections. Respiratory problems play a major role in the quality of life and life expectancy of these children [1-6].

Several medical options are available for the management of gastroesophageal reflux disease (GERD), including feeding changes, such as the elimination of cow's milk protein from the diet, or the use of food thickeners in infants, and pharmacological therapy for acid suppression: histamine-2 receptor antagonists/proton pump inhibitors and prokinetics, and/or positioning therapy [7-9]. Antireflux surgery is often considered for children with GERD who have complications or persistent symptoms and are unable to be weaned from medical therapies.

There have been reports that in neurologically impaired children, conventional drug therapy with acid suppressors or prokinetics is less effective and antireflux surgery is recommended to treat GERD symptoms [10-12]. However, surgical treatment is associated with high operative risk and is often not preferred. In addition, surgical treatment is not sufficient in some patients with recurrent pneumonia because although it effectively provides nutrition, and improves feeding-related stresses, it may exacerbate GER [13,14]. Use of thickeners is common and effective in decreasing frequent episodes of regurgitation or vomiting in infants [7-9,15,16], and in improving dysphagia in handicapped patients [17,18]. However, it remains to be investigated whether thickener is effective for GERD in neurologically impaired children.

In this study, we investigated the effects of thickening of food with two different concentrations of pectin liquid on acid exposure and symptoms that might be attributed to GER in children with cerebral palsy.

## Methods

### Subjects

We enrolled 18 patients (16 male and two female) with cerebral palsy from two hospitals, Gunma University Hospital and Gunma Rehabilitation Centre for the Physically Handicapped Children, Gunma, Japan. The average age of subjects was 11.7 ± 4.4 years old. All patients received enteral formula through a naso-gastric tube. Tracheostomy was used to treat two patients with dyspnea and wheeze, and one with laryngeal edema.

Chief complaints of patients were as follows: 12 had recurrent vomiting, six had chronic cough, and one had chronic cough and laryngitis. Recurrent pneumonias were present in four children. These symptoms were clinically suspected to be caused by GER, based on the clinical course and positive results in esophageal pH monitoring. Esophageal pH monitoring results were considered abnormal when the percentage of time that the pH is below 4.0 at the lower esophagus, i.e. reflux index, was greater than 4.0%. Clinical suspicion was formed following exclusion of other causes of these symptoms, such as organic or functional gastrointestinal disorders (for patients with vomiting) or respiratory or immunological problems (for patients with respiratory symptoms). Only two patients were of average weight. The rest were all below the third percentile. None of the patients had undergone antireflux surgery. Ten of 18 patients were given histamine-2 receptor antagonists, (Gaster; Astellas Pharma, Tokyo, Japan) as prior drug treatment for GER. We excluded patients who underwent surgical operation for GERD.

### Test enteral formula and thickener

We used an enteral formula (K4A; Q.P., Tokyo, Japan) with pectin liquid (REF-P1; Q.P.) added as a thickener. The composition of the enteral formula per 100 mL was 100 kcal, 4.5 g protein, 16 g carbohydrate, and 2.7 g lipid. The composition of the pectin liquid per 100 mL was 9 kcal, 0.2 g protein, 0.6 g carbohydrate, 0.1 g lipid, and 76 mg sodium.

Gastric contents and gas were aspirated through a nasogastric tube. After nurses injected pectin liquid through the nasogastric tube, the enteral formula was continuously injected into the stomach over 30 min.

### Measurement of formula viscosity

Enteral formula and pectin liquid were mixed and kept at 23 ± 1°C in a water bath for 5 min after being stirred 100 times. The viscosity was measured at 23 ± 1°C, at 20 rpm, with a No. 2 rotor using a Brookfield viscometer (Tokyo Keiki, Tokyo, Japan).

### Esophageal pH monitoring measurement

In the first part of this study, we performed esophageal pH monitoring over 48 hours for each subject. A single crystal antimony multi-use pH catheter (Synectics Medical, Barcarena, Portugal) with two channels [the end of the catheter, and 7 cm (5 cm for two patients under 6 years of age) above the end] was placed and connected to a portable digital data recorder (Digitrapper pH 400; Medtronic, Skovlunde, Denmark). Prior to passage, the pH catheter was calibrated at room temperature in pH 7.01 and pH 1.07 buffer solutions, according to per the manufacturer's protocol. The catheter pH electrode was passed transnasally and positioned 3 cm above the proximal margin of both diaphragms. The correct position of the catheter was confirmed by X-rays.

Subjects were randomly allocated to two groups. Patients in group A (n = 9) were fed the enteral formula including a high concentration of pectin liquid [enteral formula: pectin liquid = 2:1 (v/v)] or the enteral formula mixed with water added to a similar volume as the pectin liquid. Four subjects in group A were fed with a high-pectin diet on the first day, and with a non-pectin diet on the second day. The other five subjects in group A were fed in the reverse order. Patients in group B (n = 9) were fed with a low-pectin diet [enteral formula: pectin liquid = 3:1 (v/v)] or non-pectin diet. Treatment with acid suppressors or prokinetics, (Gasmotin; Dainippon Sumitomo Pharma, Osaka, Japan) was stopped 3 days before esophageal pH monitoring.

After recording, the single crystal antimony multi-use pH catheter was removed and data from the Digitrapper pH 400 were uploaded to a designated computer using PolygramNet software (Medtronic). The median values for the % time pH < 4 at the lower and upper esophagus, number of reflux episodes per day and duration of longest reflux episode, and number of reflux episodes longer than 5 min were analyzed. Reflux index was defined as the % time pH < 4 at the lower esophagus.

### Clinical trial

In the second part of the study, to elucidate the clinical effects of pectin liquid on GERD symptoms, four patients in group A were fed with a high-pectin diet for 4 weeks, followed by a non-pectin diet for 4 weeks. Five other patients were fed in the reverse order. Nine patients in group B were fed with a low-pectin or non-pectin diet.

During the final week of each 4-week trial period, nurses recorded the feeding volume, number of episodes of vomiting, volume of gastric residue, episodes of cough and wheeze, frequency of using oxygen for dyspnea, and the day when the child could return to school, on the special sheet that we provided. At the end of each day, the number of each event was counted and recorded on the special patient chart. High fever and episodes of bradycardia were also recorded.

To investigate in a single-blinded manner, a nurse different from the one who injected the enteral formula was on duty to record the data. Every 8 h, if the patient had cough or wheeze more than once, it was counted as 1 point. The cough-score was the sum of points during 1 week of recording. The maximum number of points was 21. Treatment with acid suppressors or prokinetics was continued during the test period.

### Ethical considerations

Informed consent was obtained from the mother of each subject. This study was approved by the Human Investigation Committee of Gunma University on 17 February 2005.

### Statistical analysis

Age and viscosity of enteral formula are reported as the means ± SD. Other variables were reported as the median value and interquartile range. Statistical significance was tested by the χ^2 ^test, unpaired Student's t test, or Wilcoxon's signed rank test, as appropriate. P < 0.05 was regarded as significant. All analyses were carried out using StatMate software (ATOMS, Tokyo, Japan).

## Results

### Formula viscosity

The viscosity of enteral formula and pectin liquid was 17 ± 1 mPa•s and 44 ± 2 mPa•s (n = 5), respectively. The viscosity of the high-pectin diet (enteral formula: pectin liquid = 200 mL: 100 mL) was 3000 ± 50 mPa•s, and that of a low-pectin content diet (enteral formula: pectin liquid = 300 mL: 100 mL) was 1200 ± 40 mPa•s (n = 5).

### Esophageal pH monitoring

**Figure 1:**
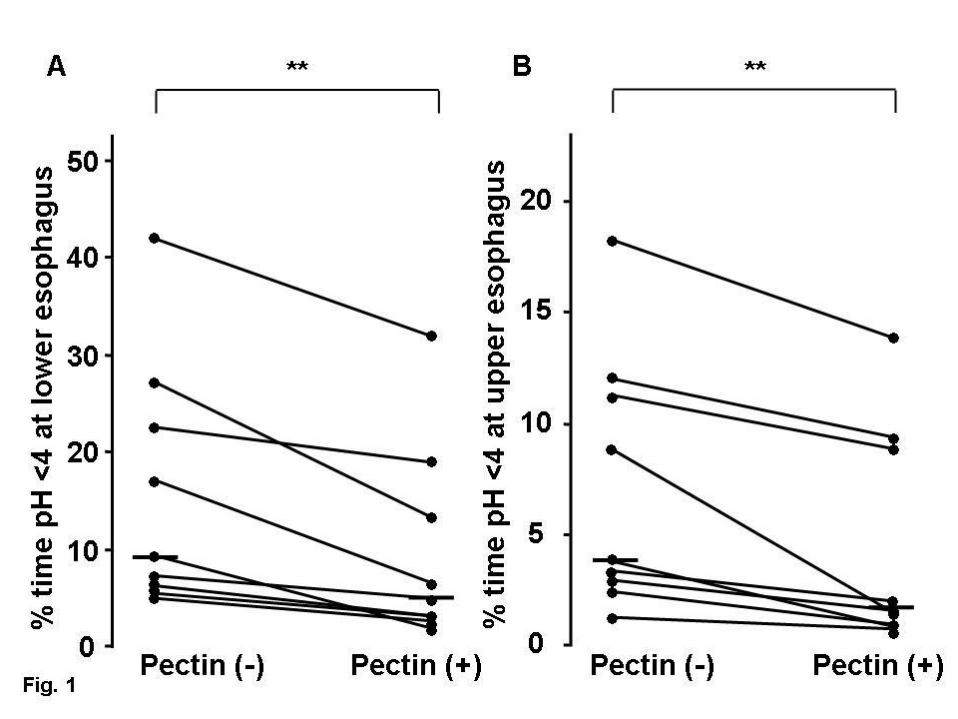
**Percent time pH < 4 at the lower esophagus (A) and % time pH < 4 at the upper esophagus (B) in patients fed with a high-pectin content diet.** Pectin (+), a high-pectin content diet. Pectin (-), non-pectin diet. Each vertical bar represents the median value. **, P < 0.01, Wilcoxon's signed rank test.

**Figure 2:**
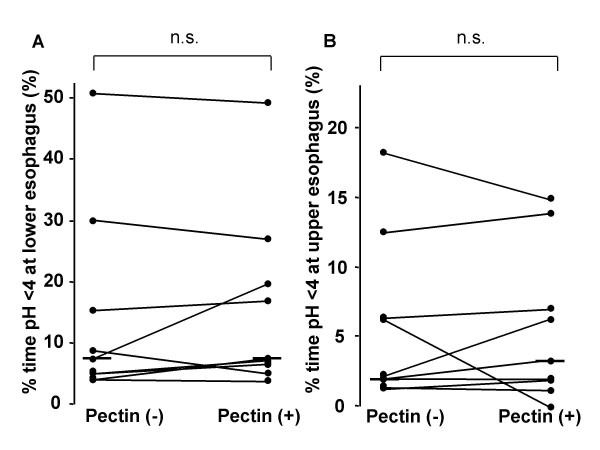
**Percent time pH < 4 at the lower esophagus (A) and % time pH < 4 time at the upper esophagus (B) in patients fed with a low-pectin content diet.** Pectin (+), a low-pectin content diet. Pectin (-), non-pectin diet. Each vertical bar represented the median value. n.s., not significant, Wilcoxon's signed rank test.

The median values for the % time pH < 4 at the lower and upper esophagus with the high-pectin diet was significantly decreased [9.2% (6.2–22.6) vs. 5.0% (3.1–13.1); P < 0.01, 3.8% (2.9–11.2) vs. 1.6% (0.9–8.9); P < 0.01 (interquartile range), non-pectin and a high-pectin, respectively] (Fig. 1A, B). Reflux index decreased by 16.4 to 81.5%. It was normalized in four of nine patients after fed with a high-pectin diet. There was no significant difference between the median values for the % time pH < 4 at the lower and upper esophagus with the low-pectin diet [7.6% (5.0–15.2) vs. 7.6% (6.7–19.7), 2.2% (2.0–6.4 vs. 3.3% (1.9–7.0), non-pectin and a low-pectin, respectively) (Fig. 2A, B). Other parameters of esophageal pH monitoring are shown in Table 1. The number of reflux episodes per day and duration of longest reflux episode were decreased significantly with the high-pectin, but not with the low-pectin diet. There was no significant difference between the number of reflux episodes longer than 5 min with and without pectin liquid.

**Table 1:** Results of esophageal pH monitoring

	Group A (high-pectin)		Group B (low-pectin)	
	Pectin (-)	Pectin (+)	Pectin (-)	Pectin (+)
Number of reflux episodes (/day)	151 (94–205)	100* (72–113)	112 (62–139)	146 (72–153)
Number of long reflux episodes (> 5 min) (/day)	4 (3–9)	3 (2–6)	1 (0–2)	3 (1–3)
Duration of longest reflux episode (min)	13 (8–46)	10* (5–26)	5 (2–23)	8 (5–21)

Values reported as the median and interquartile range.

*, P < 0.05, Wilcoxon's signed rank test

### Clinical effects

The clinical effects of pectin liquid are summarized in Table 2. The median number of episodes of vomiting decreased significantly with the high-pectin diet. There was no significant difference in the amount of gastric residue with bleeding and gastric residue > 25 mL between the two groups. However, the total gastric residue was significantly decreased by the low-pectin diet. The median cough-score was significantly decreased by both concentrations of pectin liquid. Four patients had less wheezing and the frequency of using oxygen for dyspnea was decreased in three patients when fed with pectin. There were no significant differences in the total volume of feeding and the day when the child could return to school between the groups. There were no patients with high fevers or bradycardia due to reflux episodes during the test period.

**Table 2:** Clinical effects of pectin

	Group A (high-pectin)		Group B (low-pectin)	
	Pectin (-)	Pectin (+)	Pectin (-)	Pectin (+)
Total feeding (mL/week)	6930 (5775–6970)	6950 (5775–7000)	7000 (5950–7700)	7000 (5600–7700)
Number of vomiting (/week)	2.5 (1.0–5.0)	1.0* (1.0–1.5)	0.0 (0.0–0.5)	0.0 (0.0–1.0)
Gastric bleeding (/week)	1.0 (0.0–1.5)	0.0 (0.0–0.0)	0.0 (0.0–0.0)	0.0 (0.0–0.0)
Gastric residue (> 25 mL) (/week)	3.0 (1.5–4.5)	4.0 (1.0–4.0)	2.0 (0.0–4.0)	0.0 (0.0–0.5)
Total gastric residue (/week)	4.5 (1.5–6.0)	4.0 (1.0–4.0)	2.0 (0.0–4.5)	0.0* (0.0–0.5)
Cough&wheeze (points/week)	8.5 (1.0–11.5)	2.0* (0.0–3.0)	7.0 (1.0–14.5)	1.0* (0.0–5.0)
Desaturation (/week)	3.0 (0.0–4.5)	1.0 (0.0–3.0)	0.0 (0.0–5.5)	0.0 (0.0–2.5)

Values reported as the median and interquartile range.

*, Wilcoxon's signed rank test (P < 0.05)

## Discussion

Thickening of food is commonly used for the treatment of GER in infants. Thickeners significantly decrease recurrent episodes of regurgitation or vomiting and thickening is an easy modification [7-9]. However, there have been no previous reports on the use of thickeners to decrease symptoms of GERD in children with cerebral palsy.

In our study, we first confirmed that viscosity of the enteral formula with pectin liquid increased in a dose-dependent manner. The viscosity of enteral formula was increased approximately 70-fold by the low-pectin and 180-fold by the high-pectin diet. We noted that the liquid meal changed to a semi-solid meal following addition of pectin liquid.

We found that only the high-pectin diet improved the % time pH < 4 at both the lower and upper esophagus. Reflux index decreased by 16.4 to 81.5%. It was normalized in four of nine patients. These data suggest that a semi-solid meal with added pectin decreased GER, although the effect often was partial.

We also found that both concentrations of pectin liquid had clinical effects on decreasing respiratory symptoms such as cough or wheeze, and tended to decrease the frequency of oxygen use for dyspnea, although the trends were not significant.

It is not clear why a low-pectin content diet improved chronic respiratory symptoms in patients with GERD, without improving the % time pH < 4 or reflux episodes during pH monitoring. There have been previous studies with similar results that thickened infant formula decreases regurgitation episodes, without improving reflux [15,19]. One possible reason is that only acid reflux can be detected by pH monitoring. Wenzl et al. [16] have reported the effect of thickened formula on decreasing the number of infant regurgitation episodes, based on intraluminal impedance, which suggests the importance of non-acid reflux. Another possible hypothesis is that the physiological effects of pectin on gastrointestinal motility, such as improving bowel movements [20,21], may be related to a decrease in GER-related symptoms. Further investigation is needed to determine the mechanism responsible for the relief of GERD symptoms by pectin.

Our major concern was that increasing viscosity of food by pectin liquid might cause delayed gastric emptying as previously reported [22-24]. Di Lorenzo et al. [22] have reported that pectin delayed gastric emptying and increased satiety in obese subjects, and Sandhu et al. [23] have concluded that pectin supplementation delayed gastric emptying of both liquid and solid meals in normal human subjects. We investigated the volume of gastric residue by the aspiration of residual gastric contents.

There are several techniques to measure gastric emptying, such as breath hydrogen test and scintigraphy. However, we simply evaluated gastric residue in this study to determine gastric retention after every feeding time, rather than to check gastric emptying once or twice using some physiological techniques. Our results indicated that the low concentrations of pectin liquid used in the present study did not cause delayed gastric emptying, and pectin even decreased gastric residue, which might have been because pectin liquid improved bowel motility, as previously reported [20,21].

Finally, we found that only a high-pectin diet decreased the number of episodes of vomiting. These data indicate that we may consider the use of a high concentration of pectin liquid when the effect of regular concentration of this thickener is not sufficient to decrease reflux episodes, especially for vomiting. We can evaluate the effect of pectin by means of not only improvement in clinical symptoms but also two-channel pH monitoring. We were concerned about the possibility that pectin might cause gastric retention, which was refuted even with a high concentration. However, we should also note other possible adverse effects of pectin on fat absorption and intestinal solubility or absorption of ferrous iron [25,26].

## Conclusion

Pectin liquid might improve vomiting, respiratory symptoms, and GER in children with cerebral palsy, and may be considered as an alternative therapy for GERD that can be added to pharmacological therapy.

## Competing interests

The author(s) declare that they have no competing interests.

## Authors' contributions

RM carried out esophageal pH monitoring and clinical trial, performed the statistical analysis, and drafted the manuscript. TT, HK, and NS participated in the design of the study. HA and AM participated in its design and coordination.

## Pre-publication history

The pre-publication history for this paper can be accessed here:


